# Supplementary material for: Structural and biochemical analysis of ligand binding in yeast Niemann–Pick type C1–related protein
Source: Life Sci Alliance. 2024 Oct 25;8(1):e202402990. doi: 10.26508/lsa.202402990 (PMC11512107; doi:10.26508/lsa.202402990)
Supplement: Supplementary file 1 [file LSA-2024-02990_TableS1.docx]

**Table S1.** **X-ray crystallography data collection and refinement statistics.**

| Dataset | **NCR1-NTD bound to ergosterol** | **NCR1-NTD bound to cholesterol** |
| --- | --- | --- |
| Type | Native | Native |
| **Data collection** | | |
| Space group | P 2_1_ 2 2_1_ | P 2_1_ 2 2_1_ |
| Cell dimensions  a, b, c (Å)  α, β, γ (°) | 92.76; 109.58; 151.81  90; 90; 90 | 92.03; 109.78; 151.36  90; 90; 90 |
| Monomers per asymmetric unit | 4 | 4 |
| Wavelength (Å) | 0.9686 | 0.9763 |
| Number of reflections measured  Total  Unique | 435345 (41561)*  58247 (5721) | 391689 (36093)  45554 (4305) |
| Resolution range (Å)^a^ | 45.05-2.44 (2.527-2.44) | 47.14-2.641 (2.735-2.641) |
| R_meas_ (%) | 0.09363 (1.84) | 0.1316 (2.339) |
| Mean I/σ (I) | 12.19 (0.95) | 11.39 (0.79) |
| CC½ | 99.9 (55.7) | 99.9 (45.9) |
| Multiplicity | 7.5 (7.3) | 8.6 (8.4) |
| Completeness (%) | 99.67 (99.91) | 98.86 (91.66) |
| Wilson B-factor (Å) | 69.94 | 76.56 |
| **Refinement** | | |
| Number of reflections (work/free)  R_work_ (%)  R_free_ (%) | 58103/2096 (5718/205)  21.42 (39.17)  25.72 (43.41) | 45255/4133 (2082/190)  22.05 (37.12)  25.37 (38.92) |
| Number of atoms  Protein  Ligands  Solvent | 888  1033  194 | 888  1026  193 |
| Average B-factor  Macromolecules  Ligands  Solvent | 80.94  119.20  82.53 | 85.43  129.64  83.93 |
| RMSD  Bond lengths (Å)  Bond angles (°) | 0.007  0.95 | 0.007  0.94 |
| Ramachandran statistics (%)  Favored  Allowed  Disallowed | 97.73  2.27  0.00 | 97.50  2.39  0.11 |
| Clashscore | 2.59 | 3.81 |
| Number of TLS groups | 1 | 1 |
| Deposited model PDB ID | 9F40 | 9F41 |

Values in brackets are indicative of the highest resolution shell. Only the first instance is indicated*.
